# 

**Published:** 1869-05-01

**Authors:** 


### *CONTENTS.*

ORIGINAL COMMUNICATIONS:

On the Prolonged Use of Bromide of Potassium, in Large Doses, and its value in the Treatment of Epilepsy ..... 269
Case of Acute Rheumatism—Pericarditis and Pneumonia ..... 278

TRANSLATIONS:

Regeneration of Nerves ..... 281
Medical Electricity ..... 284

EDITORIAL:

Cancer Curers ..... 297
The Western News Company ..... 297

LOOT:

Poisoning by Atropia ..... 298
Exterpation of Lachrymal Gland ..... 298
Spurious Vaccination ..... 298
Opium Culture ..... 299

